# Tree measures and the number of segregating sites in time-structured population samples

**DOI:** 10.1186/1471-2156-6-35

**Published:** 2005-06-16

**Authors:** Roald Forsberg, Alexei J Drummond, Jotun Hein

**Affiliations:** 1 Bioinformatics Research Center (BiRC) and Department of Genetics and Ecology, University of Aarhus, Arhus, Denmark; 2 Department of Zoology, University of Oxford, Oxford, England; 3 Department of Statistics, University of Oxford, Oxford, England

## Abstract

**Background:**

Time-structured genetic samples are a valuable source of information in population genetics because they provide several correlated observations of the underlying evolutionary processes. In this paper we study basic properties of the genetic variation in time-structured samples as reflected in the genealogies relating individuals and the number of segregating sites observed. Our emphasis is on "measurably evolving populations" i.e. populations from which it is possible to obtain time-structured samples that span a significant interval of evolutionary time.

**Results:**

We use results from the coalescent process to derive properties of time-structured samples. In the first section we extend existing results to attain measures on coalescent trees relating time-structured samples. These include the expected time to a most recent common ancestor, the expected total branch length and the expected length of branches subtending only ancient individuals. The effect of different sampling schemes on the latter measure is studied. In the second section we study the special case where the full sample consists of a group of contemporary extant samples and a group of contemporary ancient samples. As regards this case, we present results and applications concerning the probability distribution of the number of segregating sites where a mutation is unique to the ancient individuals and the number of segregating sites where a mutation is shared between ancient and extant individuals.

**Conclusion:**

The methodology and results presented here is of use to the design and interpretation of ancient DNA experiments. Furthermore, the results may be useful in further development of statistical tests of e.g. population dynamics and selection, which include temporal information.

## Background

### Time-structured genetic samples

Genetic samples obtained over several points in time are a valuable source of information in population genetics because they provide several correlated observations of the underlying evolutionary processes.

These time-structured samples can be separated into two qualitatively different groups. Firstly, samples may be taken over such a short evolutionary time that the occurrence of mutations between sampling points can be ignored. Samples of this type have a long standing history in the study of the process of drift and selection via observations of allele frequencies (see e.g. [1]). Secondly, time-structured samples may be obtained over intervals of evolutionary time that are long enough for mutation to become a relevant force in shaping the diversity between samples from different time points. To reflect the fact that the latter type of samples are capable of showing new variation arising, Drummond et al. used the term "Measurably Evolving Populations" (MEP) to describe populations from which biological and technological constraints allow samples of this type to be obtained [2]. Such measurably evolving populations arise from two principal sources, namely rapidly evolving microorganisms e.g. [3] and well characterised vertebrate subfossil material from which ancient DNA can be reliably amplified e.g. [4-6].

Besides a report by Nordborg [7], population genetic studies of MEPs have mostly focused on the construction and use of models for population genetic inference that incorporate the time structure of the data [8-12]. However, our interest here is not that of inference, but rather to study basic properties of time-structured samples obtained from measurably evolving populations. To this end, we use a simple model of a constant sized panmictic population of haploids and base our results on the standard coalescent process [13,14].

The paper consists of two parts. In the first part we use recursions to derive results for various measures on coalescent trees connecting time-structured samples having any time-structure. In particular, we focus on the effect that different sampling schemes have on the expected length of the branches in the tree upon which mutations can arise that are unique to the ancient individuals of the sample. This is a measure of the expected amount of genetic variation unique to the ancestral material and as such of intrinsic interest to the design of many ancient DNA studies, for example when the objective is to discover unique ancient haplotypes. In the second part, we study the case where the sample has a time-structure of only two time-points, one consisting of a number of contemporary extant individuals and one consisting of a number of contemporary ancient individuals. For this case we obtain the probability distributions of the number of segregating sites where the mutation is observed only in ancient lineages and of the number of segregating sites where the mutation is shared between ancient and extant lineages. Using these results we study the number of ancient samples needed to observe at least one unique or one shared mutation as a function of population parameters.

The results presented here should be of particular interest to studies of ancient DNA and the design of ancient DNA sampling schemes.

### Notation

**Figure 1:**
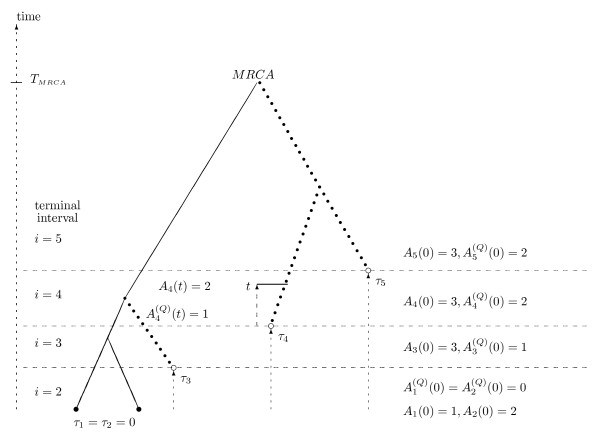
**Time-structured samples**. An illustration of a time-structured sample. Time proceeds backward as indicated by the arrow at the left. Individuals sampled at five sampling points constitutes the full sample. With each sampling point is associated a sampling time (*τ*) and the first sampling time is zero by convention. Three samples are ancient (open circles) and constitute a sub-sample *Q*. Two individuals are extant (full circles) i.e. they are both sampled at time zero (*τ*_1 _= *τ*_2 _= 0). Hence, interval one has a length of zero time units and is not depicted. The sampling intervals are numbered by *i *= 1 ... 5 and interval five is the terminal interval where after no new samples are included. At the end of the terminal interval a most recent common ancestor (MRCA) is found and this time point is denoted *T*_*MRCA*_. The total number of lineages at time *t *in interval *i *is a stochastic process with an associated variable *A*_*i*_(*t*). On the figure is given the total number of lineages at the beginning of each interval (*A*_*i*_(0)) and for interval four the number at a specific time point *t*. Likewise, the number of lineages subtending leaves exclusive to *Q *at time *t *in interval *i *is a stochastic process with an associated variable . On the figure is given the number of lineages subtending leaves exclusively in *Q *at the beginning of each interval () and for interval four the number at a specific time point *t*. Mutations occurring in ancestors which subtend leaves exclusive to *Q *(branches represented by dotted lines) will create segregating sites where the mutation is unique to the individuals in *Q *i.e. the ancient individuals.

Consider the evolution of a haploid population comprising *N *individuals. We assume selective neutrality, no recombination and a Wright-Fisher model of propagation where each individual chooses its parent independently and at random from the individuals of the previous generation [15,16]. Time is measured in units of *N *generations, and the population is assumed to remain at a constant size, which is sufficiently large that the diffusion approximation of the coalescent process applies [13]. The complete sample is produced by sampling the population serially over a sampling time-interval consisting of *n *sampling points (Figure 1) each contributing one new individual to the process. Each sampling point is associated with a sampling time (*τ*_*i*_) and hence the temporal configuration of the sample is completely determined by the ordered vector of times *τ *= (*τ*_1_,..., *τ*_*n*_) with *τ*_1 _≤ *τ*_2 _≤ ⋃ ≤ *τ*_*n*_. Sampling points and times are enumerated from the present going backwards. We let *τ*_1 _= 0 by definition, and define, *τ*_*i*,*J *_= *τ*_*i *_- *τ*_*J*_, *i *> *j*, as the difference in sampling times between sample pair *i*, *j*. Note that groups of samples may be taken at the same time so that *τ*_*k *_= *τ*_*k *__+ 1_⋃ = *τ*_*k*+*g*-1_, for a group of size *g*.

### The standard coalescent

By a continuous-time approximation, Kingman has shown that for a contemporary sample (*τ*_*i *_= 0, ∀_*i*_) a process termed the coalescent can describe the genealogical relationships of the individuals in the sample [13]. Kingman's result applies to the Wright-Fisher model and a broad class of other reproductive models that share a common set of requirements [13]. Briefly, the standard coalescent is a process describing the death of lineages through coalescence events. Some fundamental results concerning the standard coalescent which we use in the following are given here:

The waiting times *W*_*n*_, *W*_*n*-1_,..., *W*_2 _between successive coalescent events are exponentially distributed as



when time is measured in units of *N *generations [17].

Let time increase into the past and let the number of distinct line ages at time *t*, {*A*(*t*), *t *≥ 0} be given by the death process described by the coalescent. Following [18] we have that given *a *lineages were sampled at time 0, the conditional probability distribution of the number of remaining lineages at time *t *is



for 2 ≤ *b *≥ *a*, and for the case *b *= 1



where *a*_[*j*] _= *a*(*a *- 1)...(*a *- *j *+ 1), and *a*_(*j*) _= *a*(*a *+ 1)...(*a *+ *j *- 1).

In the following it will be of interest to study the number of ancestors of sub-samples which in this context are comprised of ancient lineages. Let *Q *be a sub-sample of the full sample consisting of all ancient individuals and let *Q*^*c *^be the complement of *Q *which contains the extant individuals. It is our interest to study the number of ancestral lineages at some time *t *that subtend leaves in the tree which are exclusive to *Q*, {*A*^(*Q*)^(*t*), *t *≥ 0}, as mutations occurring on these branches will be unique to *Q*, i.e. be found solely in ancient samples (Figure 1). However, this is equivalent to recording the total number of ancestral lineages at time *t*, *A*(*t*), and the number of ancestral lineages that subtend one or more leaves (not necessarily exclusively) in *Q*^*c *^{ (*t*), *t *≥ 0}, as the two are related by *A*(*t*) = *A*^(*Q*) ^(*t*) +  (*t*). The bivariate process {*A*(*t*),  (*t*)} for a sample of contemporary individuals has been studied extensively by Saunders et al. [19], and from this we extract results for the conditional distribution of the bivariate process {*A*(*t*), *A*^(*Q*) ^(*t*)} using the above relation



where *Pr*{*A*(*t*) = *b*|*A*(0) = *a*} is given by (2), and



### The coalescent process of time-structured data

When samples have time-structure (*τ*_*i *_> 0 for some *i*) the simple death process of the coalescent is replaced by a series of death processes, interrupted at specified points in time by new lineages entering the process (Figure 1). We note that this would correspond to a birth-death process of lineages if sampling events were random rather than known. However, lacking knowledge about the properties of the sampling process we restrict ourselves to condition on known sampling times. Thus, this serial coalescent process can be modelled on the basis of the standard coalescent process, by the following algorithm: At the second sampling point (no coalescence in first interval) a contemporary coalescent is initiated with two individuals; this process is continued for *τ*_3,2 _time units; at *τ*_3 _time units another individual enters the process; again a coalescent process is continued for *τ*_4,3 _time units until *τ*_4 _where yet another individual is added and so forth until *τ*_*n *_is reached and the last individual is included; from here the process continues as a standard coalescent process (as no new lineages will be added) until the most recent common ancestor (MRCA) of the sample is reached. Therefore, we refer to this last interval as the termination interval (Figure 1). The lineage number at each sample point is a stochastic variable with a distribution that must be tracked through the sampling interval. It is clear that the following results are all conditional on the temporal configuration of the sample (*τ*) and consequently this dependence is suppressed from this point.

### The mutation process

We shall be concerned with the sampling properties of nucleotide sequences. Hence, we adopt the infinitely many sites model [17], assuming that a single site experiences at most one mutational event so that every mutation that arises is represented in the sample. Furthermore, we assume that mutations are generated by a Poisson process with parameter . The compound parameter *θ *is given by *θ *= 2*N μ *where *μ *is the mutation rate per sequence per generation.

As branch lengths are measured in units of *N *generations, the expected number of segregating sites generated over a tree (or sub-section of a tree) with total branch length *l*, is .

## Results

### Measures on coalescent trees of time-structured samples

In this section we use the theory presented above to derive recursions describing various measures on coalescent trees of time-structured data.

#### Number of lineages through the sampling intervals

Let, {*A*_*i*_(*t*), 0 ≥ *t *≥ *τ*_*i*+1,*i*_} be a stochastic variable representing the number of lineages in interval *i*, *t *time units after *τ*_*i *_(see Figure 1). Consecutive death processes are related by single birth events, so that



The probability distribution of *A*_*i*_(*t*) is found by summing over all the possible lineage numbers at the start of the interval permitted by the sample configuration



Notice that the last term is given by (2), and that *Pr*{*A*_*i*_(0) = *a*}, the probability of observing a lineages at the start of the interval, is given by (5,7) and:

for *i *= 1, 2



for *i *> 2

*Pr*{*A*_*i*_(0) = *a*} = *Pr*{*A*_*i*-1 _(*τ*_*i,i*-1_) = *a *- 1}.

Lastly, let {*A*_*i*_} be the marginal lineage number in the interval [*τ*_*i*_, *τ*_*i*+1_) with probability



#### Time to the most recent common ancestor

From (1) and the above it follows that the expected time to the MRCA of a time structured sample, (*T*_*MRCA*_), is given by



#### Total branch length of the genealogy

The above results can also be applied to produce the expected total branch length for time-structured data (*B*^(*tot*)^), given by



where *B*_*i *_is the branch length added in each of the *n *- 1 time-intervals that comprise the total sampling interval,



and *B*^(*term*)^) is the branch length added over the last interval



where the latter term corresponds to the expected total branch length of the tree relating a sample of size a in a standard coalescent process [17].

#### Number of lineages subtending leaves exclusive to a sub-sample

Let the function *δ*(*Q, i*) be a membership function for the sub-set *Q *so that



Let, { (*t*, 0 ≤ *t *≤ *τ*_*i*+1,*i*_} be a death process representing the number of lineages subtending leaves exclusively in *Q, t *time units after sampling individual *i*. Consecutive death processes ( (*t*),  (*t*)) are related by single birth events, so that



The joint probability distribution over *A*_*i*_(*t*) and  (*t*) is found by summing over all the possible lineage configurations at the start of the interval that are permitted by the sample configuration and the structure of *Q*



where, , and where *Pr*{*A*_*i*_(0) = *a*,  (0) = *d*}, is given by (13,14) and (15). The unconditional probability distribution over  (*x*) is given by



Lastly, let {} be the marginal probability of the number of lineages subtending leaves exclusively to *Q *over the interval [*τ*_*i*_, *τ*_*i*+1_). Consequently,  is given by integrating over the interval length



#### The expected length of branches subtending leaves exclusive to a sub-sample

Let *B*^(*Q*) ^denote the expected total length of all branches subtending leaves only in *Q*. Similar to (10), we have that



where {} is the expected branch length added over interval *i*, given by



and the expected branch length over the last interval {} is given by



which corresponds to conditioning on the number of different lineage types present at the initiation of the termination interval and then weighing the expected branch length over the contemporary coalescent process by the probability of observing a given number of ancestors exclusive to *Q *in the individual coalescent intervals.

### Effect of sampling scheme on the expected number of segregating sites unique to ancient samples

**Figure 2:**
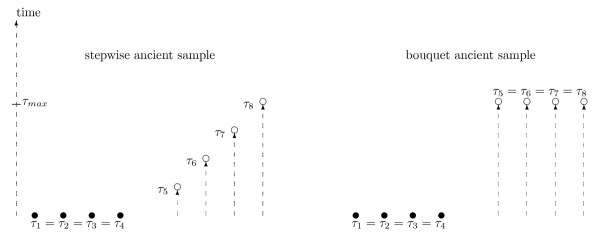
**Sample types**. The two sample types considered in the paper. The sampling time of individual *i *is indicated by *τ*_*i *_and a total of eight individuals constitute the full sample. Of these eight, four constitute a contemporary extant sub-sample (full circles) and four constitute the ancient sub-sample (open circles). In the stepwise sampling scheme the ancient samples are evenly distributed over the maximum length of the sampling interval (*τ*_*max*_) which corresponds to *τ*_8_. In contrast, the ancient samples in the bouquet sampling scheme are all taken at the maximum time attainable.

Particular constraints on studies involving ancient DNA are the maximum age at which genetic material can be obtained and the number of ancient DNA samples obtainable. The former constraint arises due to problems with dating techniques, and more importantly, with the time-dependent degradation of DNA, whereas the latter constraint arises because material containing DNA may be hard to obtain or due to financial constraints on e.g. carbon dating of fossil material. Therefore, the planning of an ancient DNA study may consist of the construction of a sampling strategy which distributes a given number of ancient samples within a fixed maximum time-interval. A possible objective of such a study may be to maximise the expected number of segregating sites where the mutation is unique to the ancient samples i.e. maximising the expected length of branches subtending leaves exclusive to the ancient samples (see equation 18). This would, for example, be the case in studies which aim at maximising the number of unique ancient haplotypes observed in order to infer past mixing events of populations such as humans and Neanderthals [6,7,20] or patterns of e.g. colonisation from an ancient population [21]. Assuming that a number of contemporary extant individuals are sampled, two strategies for sampling ancient individuals appear as natural candidates. A stepwise sampling strategy where ancient samples are distributed evenly over the available maximum time-interval and a "bouquet" strategy where all ancient samples are sampled at the maximum age (Figure 2).

**Figure 3:**
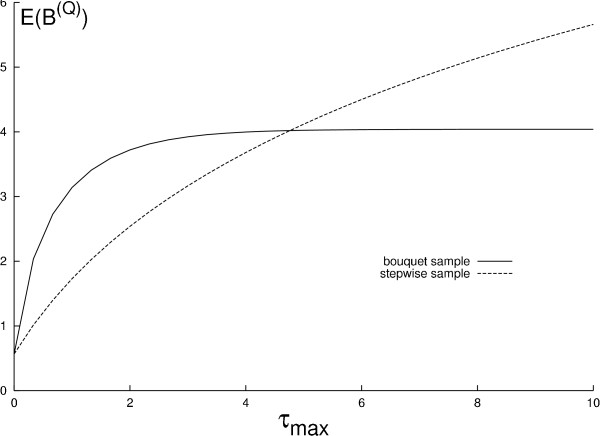
**Effect of sampling type on branch length**. The expected total length of branches subtending only ancient individuals as a function of the maximum attainable sampling time (*τ*_*max*_). The expected total length (*E*(*B*^(*Q*)^)) is shown for the stepwise and the bouquet sample types depicted in Figure 2. The number of extant individuals is 30 and the number of ancient individuals is 10. The maximum attainable sampling time is varied causing a sample type specific pattern of increase in the expected branch length (see text).

In Figure 3 we plot the expected total length of branches subtending only ancient leaves as a function of the maximum length of the total sampling interval (*τ*_*max*_) and the sampling strategy. When *τ*_*max *_is zero, lineages from the ancient sub-sample *Q *and *Q*^*c *^are interchangeable in the coalescent process and the major part of the tree will contain lineages which subtend leaves in both *Q *and *Q*^*c*^. Initially, the expected branch length for the bouquet strategy increases rapidly with increasing *τ*_*max *_since this leaves time for extant samples to coalesce within themselves before the inclusion of the ancient sample and thus interfere less with the part of the tree subtending only ancient lineages. This means that in this part of the parameter space, large gains in uniquely ancient genetic diversity can be achieved by a relatively small elongation of the sampling interval. At higher values of *τ*_*max *_the probability of observing more than one extant lineage at the time of the ancient sample is minimal and thus the expected branch length goes towards the asymptotic value of including a single extant lineage. For the step-wise strategy the initial increase in expected branch length is less marked because the ancient individuals in this case are younger and share more branches with the ancestors of the extant individuals for a given *τ*_*max*_. However, as *τ*_*max *_increases the number of surviving lineages after each sampling interval approaches one and the expected branch length asymptotes towards a linearly increasing function.

When the objective is to maximise the expected length of branches subtending only ancient lineages the bouquet strategy may intuitively seem the obvious choice as this maximises the total age of ancient material in the sample. However, Figure 3 shows that the bouquet sampling strategy only outperforms the stepwise strategy until a certain value of *τ*_*max *_is reached. Above this value it is advisable to distribute ancient samples evenly over the maximum attainable time-interval rather than to sample all ancient individuals as old as possible. This may be of particular relevance to species with small effective population sizes (*τ*_*max *_large), like top-level predators (saber-toothed tigers, lions and bears).

## The case of two sample points

In this section we focus on the simpler case with only two sampling points i.e. the bouquet sampling strategy presented above (Figure 2). Our interest is to study the distribution of segregating sites between different classes of lineages. Under the infinite sites model, the number of segregating sites in a contemporary sample of individuals has been extensively studied by [17]. Furthermore, the transient distribution of segregating sites between two time points has been studied by [22].

Suppose that a total of *n *individuals are sampled. Of these, *b *ancient individuals are sampled at the same time and constitute the sub-sample *Q *of interest. The remaining *n *- *b *individuals are all extant i.e. sampled at time 0. Assume that there are *a *ancestors left of the extant individuals at time *τ*_*n-b*_, i.e. when the ancient individuals included in *Q *are reached. Since no time passes (*τ*_*n-b*+1 _= *τ*_*n-b*+2 _= ... = *τ*_*n*_) during the sampling of the *b *lineages in *Q*, we have that  (0) = *b *and  (0) = *a*.

The coalescence process in the terminal interval now proceeds through *a *+ *b *- 1 intervals of waiting. Let *t*_*k *_denote the beginning of the *k*th coalescent interval {*k *= 0,1...*a *+ *b *- 2}.

The total number of mutations occurring on the lineages through the coalescent process in the terminal interval (*U*^(*tot*)^) will give rise to segregating sites that can be divided into three categories: mutations which occur on lineages subtending only in *Q *and which therefore create segregating sites where the mutant is unique to the ancient individuals, (*U*^(*Q*)^), mutations occurring on lineages subtending only in *Q*^*c *^causing segregating sites where the mutation is unique to the extant individuals (*U*^(*E*)^), and mutations occurring on lineages subtending in both *Q *and *Q*^*c *^giving rise to segregating sites where the mutation is shared between the ancient and the extant individuals (*U*^(*S*)^); *U*^(*tot*) ^= *U*^(*E*) ^+ *U*^(*S*) ^+ *U*^(*Q*)^. In the following we assume that the ancestral state of the genetic element under study can be accurately inferred. In the case where a segregating site represents a mutation that is found in all individuals of one group (ancient or extant) and absent from all individuals in the other group, this knowledge is required to determine whether the segregating site represents a mutation that is unique to all ancient (in *U*^(*Q*)^) or to all extant individuals (in *U*^(*E*)^).

### Segregating sites found only in ancient individuals

Through the coalescent process, lineages which subtend leaves exclusively in *Q *(ancient leaves) may die in two events: as two of these lineages coalesce, or as one of these coalesce with a lineage subtending leaves in *Q*^*c*^. Let there be ( (*t*_*k*_) = *c*) ancestors left subtending exclusively ancient leaves at the beginning of the *k*th coalescent interval.

The probability of losing a lineage subtending leaves exclusively in *Q *over the *k*th coalescent interval is



and the probability of keeping a lineage subtending leaves exclusively in *Q *over the *k*th coalescent interval is



Let  be the number of mutations unique to the lineages in *Q *that occur in the *k*th coalescent interval. Following [23] we then have the probability



The probability distribution of the number of mutations unique to the lineages in *Q *which occur from interval *k *until the MRCA {} is then given by the recursion



and the full unconditional probability of seeing *m *mutations is found by summing over all possible start values of *a*



### Segregating sites arising in terminal interval found only in extant individuals

It is evident that the probability distribution of the number of segregating sites where the mutation is unique to extant individuals can be found by exchanging *Q *and *Q*^*c *^in the above.

### Segregating sites shared between ancient and extant individuals

Segregating sites where the mutation is shared between ancient and extant samples occur on ancestral lineages that subtend leaves in both the sub-sample and the complement. Past the last sampling interval, let { (*t*), *t *≥ 0} represent the number of ancestors present at time *t *which subtend leaves in both *Q *and *Q*^*c*^. In a coalescent event, the number of lineages in  (*t*) may be reduced as two of these lineages coalesce, it may remain constant, or it may be increased by one as a lineage in  (*t*) coalesces with a lineage in  (*t*).

Given that interval *k *is initiated with ( (*t*_*k*_) = *s*) lineages subtending leaves in both *Q *and *Q*^*c *^and ( (*t*_*k*_) = *c*) ancestors left subtending exclusively ancient leaves, we have that over the *k*th interval:

The probability of losing a lineage subtending leaves in both *Q *and *Q*^*c *^is



The probability of the number of lineages subtending leaves in both *Q *and *Q*^*c *^remaining constant while a lineage subtending leaves exclusively in *Q *is lost is



The probability of the number of lineages subtending leaves in both *Q *and *Q*^*c *^remaining constant whilst the number of lineages subtending leaves exclusively in *Q *is kept constant is



and the probability of gaining a lineage subtending leaves in both *Q *and *Q*^*c *^is



Let  be the number of mutations shared between the sub-sample and the complement that occur in the *k*th coalescent interval. We then have the probability



The number of shared mutations which occur from interval *k *until the MRCA {} is then given by the recursion



and the full unconditional probability of seeing *m *mutations is found by summing over all possible start values of *a*



### Total number of segregating sites arising in terminal interval

The total number of segregating sites created in the terminal interval can be found by treating all lineages as one sub-sample in either of the recursions given above.

### Applications

**Figure 4:**
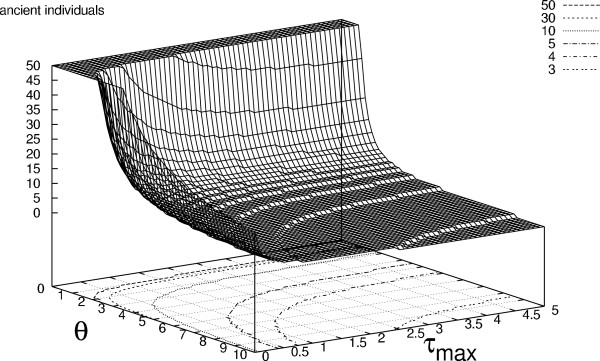
**Segregating sites unique to ancient individuals**. The number of ancient lineages required to ensure a probability higher than 0.95 of seeing at least one segregating site where the mutation is unique to ancient individuals. Samples are taken by the bouquet sampling strategy (see Figure 2) and the number of extant samples is fixed at 30. For computational reasons there is an upper limit of 50 ancient individuals. The number of lineages required is shown as a function of *θ *= 2*N μ *and the maximum attainable sampling time *τ*_*max*_. Contour lines of the required number of individuals are shown at the base of the plot.

**Figure 5:**
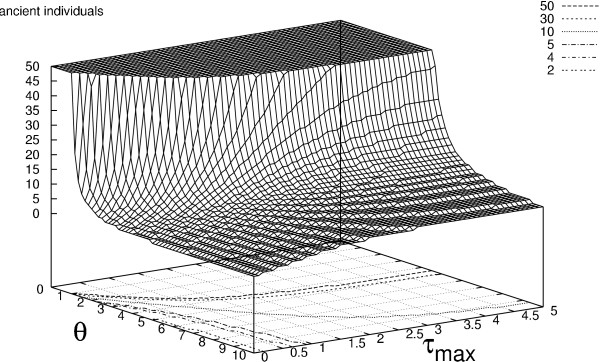
**Segregating sites shared by ancient and extant individuals**. The number of ancient lineages required to ensure a probability higher than 0.95 of seeing at least one segregating site where the mutation is shared by ancient and extant individuals. Samples are taken by the bouquet sampling strategy (see Figure 2) and the number of extant samples is fixed at 30. For computational reasons there is an upper limit of 50 ancient individuals. The number of lineages required is shown as a function of *θ *= 2*N μ *and the maximum attainable sampling time *τ*_*max*_. Contour lines of the required number of individuals are shown at the base of the plot.

In Figures 4 and 5 we plot two measures as a function of *θ *and the maximum attainable sampling time, *τ*_*max*_. The first of these is the number of ancient individuals that must be included at the second sample point to reach a probability greater than 0.95 of there being one or more segregating sites where the mutation is unique to the ancient individuals. Likewise, the latter is the number of ancient individuals that must be included at the second sample point to reach a probability greater than 0.95 of there being one or more segregating sites where the mutation is shared between ancient and extant isolates. Both are decreasing functions of *θ *which scales branch lengths in terms of mutations. At low values of *θ *the probability of seeing any mutations in the sample is low and a large amount of ancient individuals are required for any unique ancient or shared mutations to occur. The expected number of extant ancestors at the inclusion of the ancient sample decreases with *τ*_*max *_and thus the distribution of coalescent trees generated in the terminal interval becomes less dominated by lineages subtending extant leaves. As a consequence, the number of ancient individuals needed to produce any segregating sites where the mutation is unique to the ancient individuals also decreases with *τ*_*max *_(Figure 4) and the number needed to see any shared segregating sites increases with *τ*_*max *_(Figure 5). For both functions, the effect of increasing the number of extant samples can only be seen at low values of *τ*_*max *_(results not shown). This is due to the nature of the coalescent process where the coalescent intensity increases as a function of lineage number causing the additional extant lineages to coalesce before they can affect the substitution process in the terminal interval.

Typical parameter values observed in ancient DNA studies are: fragment length ~ 500 nucleotides, substitution rate per nucleotide site ~ 1·10^-7^(10^-6 ^– 10^-8 ^range for mitochondrial DNA) e.g. [4,24]. Since the parameters *θ *and *τ*_*max *_are measured in units of generations over the effective population size (*N_*e*_*), knowledge of *N*_*e *_and the maximal number of generations spanning the sampling interval (*g*_*max*_) are needed to relate these parameter values to the plots. In Table 1 we list several combinations of the parameters *N*_*e *_and *g*_*max *_and their associated values of *θ *and *τ*_*max *_along with values from Figure 4 and Figure 5. To interpret the entries in Table 1 consider that in ancient DNA studies the maximal sampling time is often in the range 30.000 ~ 50.000 years. Dependent on the generation time of the studied organism the maximal number of generations spanning the sampling interval is then given by: .

**Table 1:** The effect of sample number under different evolutionary scenarios. Result in this table are based on typical parameter values from the ancient DNA literature and a bouquet sampling scheme (see text). Fixed parameter values are: fragment length = 500 nucleotides, substitution rate per nucleotide site = 1·10^-7^, number of extant samples = 30. Given these values any combination of the population size parameter (*N*_*e*_) and the maximal number of generations spanning the sampling interval (*g*_*max *_) can be translated to a value of the population parameter (*θ*) and the maximum length of the sampling interval (*τ*_*max *_) which are measured in units of generations (see text for definition). For various such parameter combinations we here list the associated values of *θ*, *τ*_*max*_, and the calculated number of ancient samples needed to have a greater than 95% chance of seeing at least one segregating site where the mutation is unique to the ancient individuals (*n*_*u*_) and the number of ancient samples needed to have a greater than 95% chance of seeing at least one segregating site where the mutation is shared between ancient and extant individuals (*n*_*s*_).

*N*_*e*_	*θ*	*g*_*max*_	*τ*_*max*_	*n*_*u*_	*n*_*s*_
5000	0.5	1000	0.2	> 50	> 50
-	-	5000	1.0	> 50	> 50
-	-	10000	2.0	> 50	> 50
-	-	30000	6.0	> 50	> 50
-	-	50000	10.0	> 50	> 50

10000	1.0	1000	0.1	> 50	> 50
-	-	5000	0.5	> 50	> 50
-	-	10000	1.0	> 50	> 50
-	-	30000	3.0	42	> 50
-	-	50000	5.0	39	> 50

30000	3.0	1000	0.03	44	3
-	-	5000	0.17	23	5
-	-	10000	0.33	15	7
-	-	30000	1.0	9	> 50
-	-	50000	1.67	7	> 50

50000	5.0	1000	0.02	26	2
-	-	5000	0.1	16	2
-	-	10000	0.2	11	3
-	-	30000	0.6	7	6
-	-	50000	1.0	5	9

For a specific example consider the recent effort to sequence ancient human mitochondrial sequences from ~ 20.000 years ago [25]. If we assume an effective human population of ~ 10.000 and a generation time of 20 years [20], we have that for humans *g*_*max *_~ 0.1 at present. Given the population and mutation parameters chosen here, we see from Table 1 that a large number of ancient human samples (> 50) would be needed to ensure the finding of segregating sites where the mutation is unique to ancient humans. It is thus not surprising that the two ancient human mitochondrial haplotypes inferred in a study by Caramelli et al. [25] are both found to be circulating in the present day human population, and it is questionable whether unique ancient haplotypes can be obtained without a substantial elongation of either the sampling interval or the region sequenced.

## Discussion

The use of time-structured population samples has a long standing tradition in the study of rapidly evolving microorganisms. Here, the temporal component of data sets has allowed researchers to explore complex hypotheses concerning e.g. the action of selection, host-parasite co-evolution and the evolutionary response of parasites to drugs (see [2] for a review). With the advent of ancient DNA technologies it has recently become possible to obtain time-structured genetic samples that span a large number of generations from multi-cellular organisms also. As an example of the potential that such time-structured samples holds for resolving long standing questions in evolutionary biology, ancient DNA data have been used to clarify the genetic relationship between our own species and the Neanderthals [6,7], to estimate absolute rates of nucleotide substitution [4] and to infer patterns of population demography in relation to e.g. climatic changes [21].

Inspired by these technological advances, our motivation was to elaborate existing results from population genetics to the case where samples have a time-structure and to relate the results to problems faced by experimentalists.

In the first section of the paper, we have presented recursions for various tree measures and applied these to explore the effect of sampling scheme on the expected number of segregating sites where the mutation is found only in ancient individuals. Besides guiding the choice of sampling strategy, these results allow experimentalists to consider whether any genetic variation previously unseen is likely to be discovered by including ancient samples, and if so, to compare the observed number of segregating sites where the mutation is unique to ancient individuals to the expected number under a neutral model. Other applications may be relevant for the construction of time-structured data sets, depending on the objective. As an example, studies may have the objective of estimating the rate of neutral substitution. For such an estimate, substitutions occurring over the sampling interval constitute information and substitutions occurring over the terminal interval constitute noise. Thus, it is desirable to construct sampling schemes which maximise the ratio of the expected length of branches in the sampling interval over the expected length of branches in the terminal interval, and the effect of e.g. sampling schemes on this measure could also be explored using the results from this paper.

In the second part of the paper we studied the simpler case where samples are taken at only two time-points. This simplification allowed us to present results concerning the full probability distribution of the number of segregating sites where the mutation is unique to the ancient individuals and of the number of segregating sites where the mutation is shared between ancient and extant individuals. As an application of these we have shown the number of ancient lineages needed for a sample to have a high probability of showing at least one segregating site where the mutation is unique to the ancient material or at least one segregating site where the mutation is shared between ancient and extant material. These results should be of interest to experimentalists who wish to evaluate the amount of information likely to be obtained from a given time-structured sample.

In the applications presented we have focused on moderate sample sizes. For large samples (>> 50) computational problems arise due to the computational load of the recursions and problems with computer representation of very small probabilities. If larger sample sizes are to be considered these problems must be circumvented. This could be done by simplification of recursions, the use of approximative calculations and the derivation of probabilities through Monte Carlo procedures [7]. Smaller sample sizes may, however, be sufficient to extract general properties as the effect of increasing sample size on e.g. total branch length in the coalescent quickly diminishes.

The results concerning tree measures are applicable to all time-structured samples. However, the results on segregating sites are derived under the assumption of the infinite sites model which is invalid for data sets taken over long time-intervals from rapidly evolving genetic elements such as the mito-chondrial control region or genomic regions of RNA viruses.

For simplicity we have only considered the case of a constant population, but if the demographic function is known, the effect of varying population size could be accommodated into the tree measure recursions given here via the generalisation of (2) given in [26]. This is also true for the tree measures concerning sub-samples, since the result in (4) is purely combinatorial and thus independent of the demographic function. For the results concerning the number of segregating sites, an extension to varying population size is not possible with the present approach as it relies on the independence between consecutive coalescent intervals.

## Conclusion

The focus of this paper has been on sampling issues and the production of results that may be of use in the design and interpretation of experiments including time-structured genetic samples, particularly ancient DNA experiments. However, given that the inclusion of time-structure increases the statistical power in evolutionary inference, it is our hope that the results presented may also be useful in the pursuit of statistical tests, in the flavour of Tajima's D [27], which are applicable to time-structured samples.

## Authors' contributions

RF conceived the idea for this manuscript, provided the majority of the results, implemented results into computer code, performed the experiments and wrote the manuscript. AJD helped in the discussion of results, the implementation into software and the design of experiments. JH aided in conceiving the idea for the manuscript, discussing results and providing mathematical results. All authors read and approved the final manuscript.
